# Measuring Clinical Learning Environment Across Three Residency Programs Using a Postgraduate Hospital Educational Environment Measure (PHEEM) Scale at a Tertiary Care Hospital in the United Arab Emirates

**DOI:** 10.7759/cureus.99422

**Published:** 2025-12-16

**Authors:** Nouf AlBisher, Fayeza AlAmeri, Dolhyt Detera, Humariya Heena

**Affiliations:** 1 Family Medicine, Zayed Military Hospital, Abu Dhabi, ARE; 2 Medical Education, Zayed Military Hospital, Abu Dhabi, ARE; 3 Medical Education and Training, Zayed Military Hospital, Abu Dhabi, ARE

**Keywords:** clinical learning environment, perceptions of role autonomy, perceptions of teaching, postgraduate hospital educational environment measure (pheem) scale, postgraduate medical education

## Abstract

Background

The clinical learning environment for hospital trainees needs evaluation for patient safety and healthcare quality to acquire theoretical knowledge, clinical skills, and problem-solving abilities.

Objective

We evaluated participants from three residency programs at a teaching hospital as per the PHEEM questionnaire domains to understand the learning environment and areas of improvement in the program.

Methods

After study approval, a cross-sectional study was conducted from December 2023 to March 2024 with a validated questionnaire, which was piloted and administered online to 51 participants with slight modifications for cultural inclusivity and relevance and also checked for reliability and validity. The response rate was 100% for all questions. Data analysis was conducted using SPSS Statistics Version 22.0 at p < 0.05.

Results

A total of 51 participants completed the questionnaire, and the majority of the trainees were males and emergency medicine residents. Perceptions of role autonomy and perception of teaching had a mean score of 38.7 (out of 52) and 45.9 (out of 60), respectively, indicating a generally positive outlook, while perception of social support had a mean score of 33.0 (out of 44). The total mean study score was 117.7 out of 156. The mean scores for all items ranged from 2.51±1.03 to 3.31±0.58. Areas for improvement included collaborating with other doctors who are in the same year. The results revealed no significant variations in the scores across different strata. There was a slightly higher perception of social support among female students.

Conclusion

This study revealed a positive perception and outlook toward various domains. There is a need to provide counseling services for a better interpersonal environment at work.

## Introduction

The Accreditation Council for Graduate Medical Education International (ACGME-I) has mandated the Clinical Learning Environment Review (CLER) to assess the quality of education and provide a clinical learning environment (CLE) to trainees to enhance patient safety and healthcare quality in accredited institutions through the Next Accreditation System. In addition, its focal areas include applying theoretical knowledge to practice, acquiring clinical skills, and developing problem-solving and clinical reasoning skills [1]. Published reports suggest that CLER contributes to enhanced learning, performance, satisfaction, and success, but it can also lead to trainee burnout if perceived negatively [2].

The importance of nurturing a favorable CLE cannot be overemphasized, as it contributes to efficient learning, professional development, the quality of patient care, and the institution's overall performance. Various studies have indicated that a favorable CLE positively links to enhanced on-the-job learning, improved test scores, and heightened career satisfaction, while an adverse outcome has been linked to trainee fatigue and decreased patient care quality. Nevertheless, the complexity of the CLE needs continuous assessment, regular evaluation, and focused interventions to guarantee an atmosphere that supports efficient learning and professional development [3]. Prioritizing the continuous appraisal and improvement of the CLE remains vital for preserving a supportive and enriching educational environment that encourages the advancement and well-being of both learners and the broader medical establishment [4].

In recent times, CLE in teaching hospitals has received considerable attention, with greater emphasis on ensuring duty hours’ adherence, on-call room availability, and fatigue management [5]. The Directorate of Health is the apex governing authority for medical education in the Emirate of Abu Dhabi. There are at least eight medical schools in the whole of the UAE, and despite a large number of hospitals offering different residency programs in the Emirate of Abu Dhabi, only a handful of studies have surveyed the CLE. To date, PHEEM as an instrument to assess CLER has been used by one study conducted in Dubai, while another one in Abu Dhabi assessed the learning environment using CLER focal area questions only [5,6].

The UAE’s multicultural society and gender norms present both challenges and opportunities for residency training. While cultural diversity enhances exposure and competence, it also necessitates structured approaches to overcome communication and expectation gaps. Similarly, gender norms must be carefully navigated to ensure equitable clinical exposure for both male and female residents [7].

Our study is one of the few to employ the PHEEM scale to measure the CLE in three residency programs in the United Arab Emirates (UAE) and compare perceptions of the CLE by gender, level of training, and work experience. The study uses the three domains of PHEEM to shed light on the perceptions and highlight the strengths and opportunities for improving the residency programs.

This article was previously posted to the Research Square preprint server on July 12, 2024.

## Materials and methods

From December 2023 to March 2024, 51 residents from three specialization streams at Zayed Military Hospital in Abu Dhabi participated in a cross-sectional study. Zayed Military Hospital is a 330-bed hospital located in the heart of Abu Dhabi, accredited for three specialization programs: internal medicine, emergency medicine, and family medicine. Each year, the faculty identifies the objectives of CLER focus areas through periodic meetings, along with an assessment of areas for improvement. Clear-cut benchmarks are set to provide residents with the best possible education and training. This study used an internationally validated self-administered questionnaire (PHEEM) [8]. The three major domains of PHEEM were assessed: perception of autonomy, perception of teaching, and perception of social support. We modified the 40-item questionnaire as follows: Under the question “There is racism in this post,” the following sub-questions were added to clarify the meaning in the context of cultural inclusivity in the UAE. “There is equal opportunity regarding the annual bonus,” “Equal opportunities are provided for electing the president of the resident association,” and “Equal opportunities are provided regarding other benefits.”

Furthermore, considering the distinct social and cultural norms or expectations of behavior in the UAE, the following sub-questions were added to the questions: “There is sex discrimination in this post,” “Consideration is given to females for them to avoid areas requiring physical strength,” and “Females are treated differently due to cultural norms here.”

The modified questionnaire was validated by experts. As per the guidelines, a committee of 2 experts, each in research methodology and health professionals' education, further confirmed the validity of the questionnaire with Cronbach’s alpha >0.70. We conducted a pilot study on 10 residents from three specialties who answered the questionnaire without any difficulties.

Before distributing the questionnaire, consent forms were sent to the participants, detailing the study procedures. Residents who consented to participate in the study were sent the questionnaire via Microsoft Forms, a web-based application that allows users to create and distribute surveys. This method was chosen to facilitate the questionnaire's completion and minimize the time required for data collection. To protect the privacy of the participants, no identifying information, such as registration numbers or names, was collected. We sent two reminders to participants who had not completed the survey after the initial distribution. The results of the survey were recorded in an Excel spreadsheet. Participants were instructed to read each statement carefully and to respond using a five-point Likert scale ranging from "strongly agree" to "strongly disagree." It was emphasized that each participant should apply the items to his or her current learning situation and respond to all 40 questions. The PHEEM scale was scored as follows: 4 points for strongly agree (SA), 3 points for agree (A), 2 points for uncertain (U), 1 point for disagree (D), and 0 points for firmly disagree (SD). However, four of the 40 items (numbers 7, 8, 11, and 13) were reverse-scored, meaning that 0 points were assigned for SA, 1 point for A, 2 points for U, 3 points for D, and 4 points for SD. The 40-item PHEEM has a maximum score of 160, indicating the ideal educational environment as perceived by the resident. A score of 0 is the minimum and would be a distressing result for any medical educator. The overall score of 81-120 was considered more positive than negative, but there was room for improvement. You can also use the PHEEM to identify specific strengths and weaknesses within the educational climate. Items that have a mean score of 3.5 or over are real positive points. Any item with a mean of 2 or less should be examined more closely, as it indicates problem areas. Items with a mean between 2 and 3 are aspects of the climate that could be enhanced. The Research and Ethical Committee of Zayed Military Hospital approved the study. All data were gathered in Microsoft Excel (Microsoft Corp., Redmond, WA) and analyzed in IBM SPSS Statistics Version 22.0 (IBM Corp, Armonk, NY, USA). The statistical significance for all calculations was set at p < 0.05. We calculated the frequencies and percentages for all items in the questionnaire, as well as the mean and standard deviation for the continuous variables. Scores from 0 to 4 were assigned from the 5-point Likert scale PHEEM questionnaire responses. The scores were reversed because, in the negative questions, for example, for a "strongly agree" answer, the score has to be reversed as compared to normal questions. Normality tests for the three subscales and global scores were conducted, whereas in the absence of them, Kruskal-Wallis and Mann-Whitney U tests were conducted to see the association between participants' demographics and the PHEEM scale scores. The ethical approval was obtained from the Ethics and Research Committee at Zayed Military Hospital, Abu Dhabi, UAE, under approval number 2023.25.

## Results

**Figure 1:**
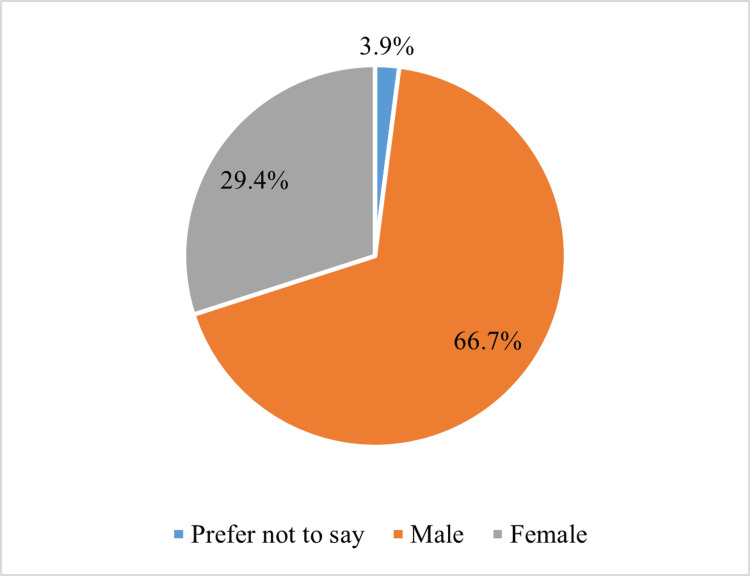
Distribution of gender

The response rate was 100% for all questions for all participants (51). The majority of respondents identified as male (66.7%, n=34), followed by female (29.4%, n=15), with a small portion preferring not to disclose their gender (3.9%, n=2) (Figure 1). The most common age range among respondents was 26-35 years (n=40, 78.4%). The highest frequency of respondents belonged to post-graduate year (PGY) 2 and PGY3 (23.5%, n=12). Emergency medicine was the most frequently reported specialty among respondents (33.3%, n=17) (Table 1).

**Table 1:** Demographic data of the participants *Graduated residents PGY, post-graduate year

		No.	%
Gender	Male	34	66.7
	Female	15	29.4
	Prefer not to disclose	2	3.9
Age (years)	18-25	5	9.8
	26-35	40	78.4
	36-45	6	11.8
Year	PGY1	9	17.6
	PGY2	12	23.5
	PGY3	12	23.5
	PGY4	6	11.8
	Other	12	23.5
Specialty	Emergency	17	33.3
	Family medicine	16	31.4
	Internal medicine	16	31.4
	Other*	2	3.9

PHEEM scale scores

Scores for all items in the PHEEM questionnaire were calculated, and the results were interpreted as a more positive learning environment than a negative one (Table 2). Among the key findings, perceptions of role autonomy stood out with a mean score of 38.7 out of a maximum of 52, indicating a generally positive outlook towards job autonomy. The outcome suggests that individuals feel empowered and in control over their roles, fostering a conducive work environment. Similarly, perceptions of teaching received a notably high score of 45.9 out of 60, which reflects a positive trajectory in teaching experiences. However, there is room for enhancement in perceptions of social support, with a mean score of 33.0 out of 44, suggesting a need for strengthening interpersonal connections and support networks within the professional setting. Overall, while the total mean study score of 117.7 out of 156 indicates a predominantly positive atmosphere, it also underscores areas for improvement.

**Table 2:** PHEEM scale scores PHEEM, Postgraduate Hospital Educational Environment Measure

PHEEM subscales	Mean (SD)	Max score	Interpretation based
Perceptions of role autonomy	38.7 (5.5)	52	A more positive perception of one's job
Perceptions of teaching	45.9 (6.8)	60	Moving in the right direction
Perceptions of social support	33.0 (4.9)	44	More positive than negative
Total PHEEM score	117.7 (15.9)	156	Is more positive than negative but room for improvement

The overall means ± SDs for all items in the PHEEM questionnaire are given in Table 3. The mean for all items ranged from 2.51±1.03 to 3.31±0.58. The lowest-rated score was for item 16, I have a good collaboration with other doctors in my same year (2.51±1.03), and the highest-rated score was for item 20, This hospital has good-quality accommodation for junior doctors, especially when on call (3.31±0.58).

**Table 3:** PHEEM domains for all items PHEEM, Postgraduate Hospital Educational Environment Measure; SpR, speciality registrar

Item	Mean	±SD
Perceptions of role autonomy		
Q1: I have a contract of employment that provides information about hours of work	3.00	0.80
Q4: I had an informative induction program	3.13	0.69
Q5: I have the appropriate level of responsibility in this post	3.25	0.52
Q8: I have to perform inappropriate tasks	3.02	0.93
Q9: There is an informative junior doctors’ curriculum handbook	2.74	0.92
Q11: I am bleeped inappropriately	2.60	1.12
Q14: There are clear clinical protocols in this post	2.96	0.86
Q17: My hours conform to what is required in internship guidelines	2.76	0.79
Q18: I have opportunity to provide continuity of care	3.16	0.70
Q29: I feel part of a team working here	3.18	0.56
Q30: I have opportunities to acquire the appropriate practical procedures for my grade	2.96	0.72
Q34: The training in this post makes me feel ready to be an SpR/consultant	3.04	0.69
Q40: My clinical teachers promote an atmosphere of mutual respect	3.18	0.48
Cumulative scores of the above items out of 52 (mean)	38.70	
Perceptions of teaching		
Q2: My clinical teachers set clear expectations	3.08	0.59
Q3: I have protected educational time in this post	3.02	0.68
Q6: I have good clinical supervision at all times	3.12	0.71
Q10: My clinical teachers have good communication skills	3.16	0.61
Q12: I am able to participate actively in educational events	2.96	0.77
Q15: My clinical teachers are enthusiastic	3.10	0.64
Q21: There is access to an educational program relevant to my needs	2.98	0.68
Q22: I get regular feedback from seniors	3.12	0.68
Q23: My clinical teachers are well organized	3.08	0.66
Q27: I have enough clinical learning opportunities for my needs	3.00	0.66
Q28: My clinical teachers have good teaching skills	3.08	0.63
Q31: My clinical teachers are accessible	3.06	0.73
Q33: Senior staff utilize learning opportunities effectively	2.98	0.71
Q37: My clinical teachers encourage me to be an independent learner	3.18	0.48
Q39: The clinical teachers provide me with good feedback on my strengths and weaknesses	3.12	0.55
Cumulative scores of the above items out of 60 (mean)	45.90	
Perception about social support		
Q40: There is racism in this post	3.23	0.97
Q13: There is sex discrimination in this post	3.23	0.86
Q16: I have good collaboration with other doctors in my same year	2.51	1.03
Q19: I have suitability access to careers advice	3.00	0.49
Q20: This hospital has good quality accommodation for junior doctors, especially when on call	3.31	0.58
Q24: I feel physically safe within the hospital environment	2.96	0.75
Q25: There is no-blame culture in this post	2.78	0.94
Q26: There are adequate catering facilities when I am on call	2.98	0.68
Q35: My clinical teachers have good mentoring skills	2.92	0.84
Q36: I get a lot of enjoyment out of my present job	3.22	0.97
Q38: There are good counseling opportunities for junior doctors who fail to complete their training satisfactorily	2.94	0.68
Cumulative scores of the above items out of 44 (mean)	33.03	

The items with mean scores between 2.5 and 3 in the provided dataset signify responses indicating moderate agreement or neutrality regarding various aspects of the professional environment. Notably, these responses neither strongly affirm nor negate the statements presented. For instance, respondents express a middling level of collaboration with peers in the same year (Q16) and occasional instances of inappropriate bleeping (Q11). Additionally, perceptions regarding the availability of an informative junior doctors’ curriculum handbook (Q9) and adherence to internship guidelines regarding working hours (Q17) also fall within this moderate range of agreement. Moreover, the presence of a no-blame culture within the professional setting (Q25) and the effectiveness of clinical teachers' mentoring skills (Q35) evoke moderate levels of respondent agreement. Furthermore, statements about the existence of clear clinical protocols (Q14), opportunities for active participation in educational events (Q12), and access to relevant educational programs (Q21) similarly garner moderate levels of agreement. Additionally, respondents express moderate levels of assurance regarding the physical safety within the hospital environment (Q24) and the utilization of learning opportunities by senior staff (Q33). Moreover, the availability of adequate catering facilities during on-call duties (Q26) also falls within this moderate range of agreement. These findings underscore the nuanced perceptions held by respondents, indicating neither overt positivity nor negativity but rather a balanced stance or potential uncertainty in their evaluations.

In addition to the PHEEM scale, two modified questions explored the participants’ opinions on racism and sex discrimination. Few participants (15.7%, n=8) strongly disagreed that there are equal opportunities regarding yearly bonuses, opportunities to elect the resident association president, and opportunities to benefit from other benefits. Furthermore, participants also agreed (9.8%, n=5) that consideration is given to women to avoid physically strenuous areas and that females are treated differently due to cultural norms here. In comparison, few others strongly disagreed (7.8%, n=4) and disagreed (5.9%, n=3) with the same. We employed the Mann-Whitney U and Kruskal-Wallis H tests to analyze the differences in PHEEM scores across gender and year of study. The analysis concluded that there were no statistically significant differences in the scores of PHEEM subscales or global scores across genders and years of study. The mean score of perception of social support was higher among female students (34.1±5.9) compared to males (32.7±4.3), with p=0.08. The total mean score was also observed to be higher in females (120.6±19.0) than in males (117.2±14.4), p=0.422 (Table 4).

**Table 4:** Gender scores for different PHEEM subscales PHEEM, Postgraduate Hospital Educational Environment Measure

PHEEM subscales	Mean (SD)		Mann-Whitney U	p-Value
	Female	Male		
Perceptions of role autonomy	39.7 (5.3)	38.6 (5.4)	236.5	0.684
Perceptions of teaching	46.7 (8.3)	45.8 (6.3)	237.5	0.703
Perceptions of social support	34.1 (5.9)	32.7 (4.3)	176	0.085
Total PHEEM score	120.6 (19.0)	117.2 (14.4)	218	0.422

The mean score for all PHEEM subscales and the global score were observed to be higher among PGY4 students than among the other year groups, although they were not statistically significant (Table 5). The results from Table 5 also suggest very small to small effect sizes with wide 95% CIs. This suggests that there are no meaningful differences across PGY groups for the PHEEM subscales in our sample.

**Table 5:** Year-wise score for different PHEEM subscales with 95% confidence intervals. *Graduated residents PHEEM, Postgraduate Hospital Educational Environment Measure

PHEEM subscales	Mean (SD)					Kruskal-Wallis H	p-Value	η² (effect size)	95% CI for η²
	PGY1	PGY2	PGY3	PGY4	Other*				
Perceptions of role autonomy	38.8 (5.9)	38.1 (6.3)	37.5 (5.9)	42.6 (5.1)	38.5 (4.1)	4.304	0.366	0.006	-0.07 to 0.14
Perceptions of teaching	45.6 (7.5)	46.9 (5.2)	44.4 (8.3)	51.1 (6.3)	44.1 (5.9)	6.048	0.196	0.041	-0.07-0.14
Perceptions of social support	32.8 (5.6)	32.0 (4.3)	31.5 (6.1)	36.0 (5.5)	34.0 (2.6)	5.295	0.258	0.026	-0.07-0.14
Total PHEEM Score	117.4 (18.3)	117.1 (14.0)	113.5 (19.3)	129 (15.6)	116.8 (11.1)	4.442	0.349	0.009	-0.07-0.14

## Discussion

The residency program in the UAE follows the ACGMIE guidelines along with other boards. Our hospital, an exclusive military hospital for the UAE, has a relatively young residency program. The ACGMIE regularly assesses the postgraduate training facilities to uphold training standards and accredit junior doctors for ongoing training. However, local ways of administering programs or perceptions of the faculty might contribute to differences in the results between the US and the UAE. Furthermore, programs in community-based hospitals may show different results from ours.

In this study, we tried to understand the perceptions of trainees and other environmental components that impact their learning experience to determine where improvements could be made. The residents constantly struggle with their work at the hospital, which involves caring for sick and dying patients, and this takes an emotional toll on them while they also pursue their academics and research. On top of it, they have family and social contacts to take care of, more so for females. Therefore, the quality of the learning environment in a teaching hospital significantly contributes to providing solace and comfort to the residents through an explicit curriculum and goals.

Roff constructed and validated PHEEM, a tool with excellent reliability globally, with Cronbach's alpha ranging from 0.84 to 0.95 [9]. The response rate and reliability of PHEEM in our study were high, which is in agreement with other studies [10].

Our perceptions were mostly positive, not negative, albeit with a few elements of concern stemming from the strong disagreement among a few participants about equality in yearly bonuses, resident elections, and other benefits. However, there was general agreement regarding the consideration given to females due to the cultural norms prevalent here.

The residents are more considerate toward work and learning balance, as indicated by their responses to questions regarding role autonomy. The overall score is higher, similar to other studies in the developing world, probably due to experienced teaching staff and variation in resident workload [11-14].

In contrast, studies conducted in the African continent report a very low score of 80 or lower due to an apparent lack of healthcare facilities, policies, curriculum for residents, and implementation in practice [15,16]. Unusually, studies from the region showed a very low overall score of below 80, indicating plenty of problems in the residency program [17-20].

High PHEEM scores are associated with better knowledge and exam performance [21]. The overall maximum scores indicated no significant issues in the learning environment. In this study, the items that received lower mean values included collaboration with other doctors in the same year, inappropriate bleeping, adherence to internship guideline hours, and the availability of a handbook for doctors. The participants in this study did not feel the same about significant issues affecting a positive learning environment, such as long working hours, unavailability of clinical protocols, inefficient use of training time, lack of constructive feedback, and presence of a blame culture, as in other similar studies [22].

The low item points identified in our study are not difficult to fix, such as providing handouts, which are management issues. However, inappropriate bleeping can lead to a stressful work environment, affecting a conducive learning environment and requiring appropriate behavioral measures.

Some of the items on the PHEEM questionnaire in our study received average scores of more than 2, which are far better than other studies, where most items received response scores below 2 on average [23,24]. In contrast, the majority of responses exceeded a mean value of 3, indicating that our residents were generally satisfied with various aspects of the learning environment across all specialties. These areas included the availability of adequate catering facilities, physical safety within the hospital environment, and utilization of learning opportunities by the senior staff. However, there is scope for improvement.

Our study reported no difference in gender responses, but the mean score and the total score for social support in females were higher than in males, in contrast to another study in the region [25]. Grech found a significant difference in perception between the genders, and like our study, females had a better perception score than males [26]. This disparity can be explained by the fact that culture can affect the perceptions between males and females.

In other studies, residents reported difficulties accessing their supervisors, which contradicts the findings of our study. Residents are provided with appropriate supervision, feedback, and mentoring support. This improvement is due to the introduction of constant faculty development programs, including those for improving academic teaching skills.

Our study showed that for PHEEM scores by level of training, senior trainees had better perceptions of the CLE, which is in agreement with Khoja, as residents learn to adapt to the work stressors over time and are in a better position to evaluate their residency experience. However, other studies do not find changes in perception about the year of residency [27-29].

Hence, a longitudinal study can provide a better outlook on this aspect. Our residents could also be provided opportunities to join renowned international medicine societies and present their research at international conferences and meetings.

Our study had limitations, the major one being a cross-sectional study on a very small sample of residents, as the residency programs in this hospital accept a limited number of doctors due to a rigorous selection process. Nevertheless, our study was a useful insight into how the learning environment at a small teaching hospital met international standards. The online distribution of the questionnaire with reminders led to an excellent response rate, as we had a 100% response rate from the participants with completed questionnaires. All our respondents were proficient in English, and the chance of misunderstanding the questions was consequently very low.

## Conclusions

Our study is one of the few in the UAE that identifies the interpersonal and gender issues related to learning environments with a higher perception of social support among females. The positive learning outcome of our study provides an impetus for more youth, especially females in this country, to pursue their medical residency at our hospital. Besides, curriculum planners can learn specific approaches required to enhance the quality of residency educational programs in this country. We also believe that having hands-on educational material available during resident onboarding and freely available counseling services are essential for better interpersonal relationships between residents.
